# Globin mRNA reduction for whole-blood transcriptome sequencing

**DOI:** 10.1038/srep31584

**Published:** 2016-08-12

**Authors:** Kaarel Krjutškov, Mariann Koel, Anne Mari Roost, Shintaro Katayama, Elisabet Einarsdottir, Eeva-Mari Jouhilahti, Cilla Söderhäll, Ülle Jaakma, Mario Plaas, Liselotte Vesterlund, Hannes Lohi, Andres Salumets, Juha Kere

**Affiliations:** 1 Department of Biosciences and Nutrition, Karolinska Institutet, Huddinge, Sweden; 2 Competence Centre on Health Technologies, Tartu, Estonia; 3 Molecular Neurology Research Program, University of Helsinki and Folkhälsan Institute of Genetics, Helsinki, Finland; 4 Institute of Molecular and Cell Biology, University of Tartu, Tartu, Estonia; 5 Department of Women’s and Children’s Health, Karolinska Institutet, Stockholm, Sweden; 6 Department of Reproductive Biology, Estonian University of Life Sciences, Tartu, Estonia; 7 Institute of Biomedicine and Translational Medicine, University of Tartu, Tartu, Estonia; 8 Department of Obstetrics and Gynaecology, University of Tartu, Tartu, Estonia; 9 Department of Obstetrics and Gynecology, Helsinki University Hospital, Helsinki, Finland

## Abstract

The transcriptome analysis of whole-blood RNA by sequencing holds promise for the identification and tracking of biomarkers; however, the high globin mRNA (gmRNA) content of erythrocytes hampers whole-blood and buffy coat analyses. We introduce a novel gmRNA locking assay (GlobinLock, GL) as a robust and simple gmRNA reduction tool to preserve RNA quality, save time and cost. GL consists of a pair of gmRNA-specific oligonucleotides in RNA initial denaturation buffer that is effective immediately after RNA denaturation and adds only ten minutes of incubation to the whole cDNA synthesis procedure when compared to non-blood RNA analysis. We show that GL is fully effective not only for human samples but also for mouse and rat, and so far incompletely studied cow, dog and zebrafish.

As a type of liquid biopsy, blood is widely used in clinical research due to its ease of sampling, its transport of biomolecules from all over the body and its rapid dynamics. Blood carries proteins, metabolites and cells, and cells such as erythrocytes, leukocytes and platelets can provide genomic DNA, microRNA and mRNA as biomarkers for a number of applications^1^,^2^,^3^. Using whole-blood RNA (wbRNA) for expression profiling by RNA-sequencing (RNA-seq) involves challenges such as a low proportion of leukocytes (4–11 × 10^6^/l) and a high prevalence of erythrocytes (4–5 × 10^9^/l), resulting in a significant amount of globin mRNAs (gmRNA) from erythrocytes. As a result, the wbRNA pool is enriched with gmRNA to the extent that 50–80% of the wbRNA-seq reads map to gmRNA^4^,^5^. This high prevalence of gmRNA complicates transcriptome studies of wbRNA, as the massive conversion of gmRNA molecules to globin cDNA (gcDNA) hijacks a significant portion of cDNA synthesis power while simultaneously leaving biologically relevant molecules undetectable. The available gmRNA reduction methods for RNA-seq, including GLOBINclear^TM^ (ThermoFisher) and Globin-Zero^TM^ (Illumina), help overcome this limitation but also add more hands-on steps, thereby compromising the quality of the RNA (reviewed in Supplementary Fig. 1).

In adult humans, highly expressed gmRNA alpha 1 and 2 (*HBA,* hereafter α) and beta (*HBB*, β) gmRNAs are unusually stable to ensure continuous hemoglobin protein expression in differentiated erythrocytes^6^. The cellular stability of gmRNA is explained in part by a closed loop structure, a characteristic shared by almost all eukaryotic mRNAs. Specifically, the gmRNA contains a cis-acting sequence, C-rich element in its 3′ untranslated region, and a 5′ m^7^ Gppp cap structure, which are critical for the stability of this long-lived mRNA^7^,^8^.

To reduce the abundance of gmRNA in gene expression-based studies, we systematically evaluated a number of gmRNA-specific oligonucleotides (GlobinLock, GL) that prevent or at least significantly reduce the priming of gcDNA synthesis. Our GL approach reduces the negative effects of gmRNA presence in expression studies and eliminates the need for additional pipetting. For adult human with dominating gmRNA α and β, a single pair of specific oligonucleotides in RNA denaturation buffer is used. The GL is effective immediately after RNA denaturation by binding to gmRNA molecules at 3′ site and specifically blocking the binding site of oligo-T primer. The GL mediated gmRNA reduction procedure adds only ten minutes of incubation to the cDNA synthesis procedure.

## Results and Discussion

We observed that oligonucleotide hybridization with gmRNA masks the binding site of the anchored oligo-T primer (T_30_VN, oligonucleotides are shown in Supplementary Table 1), suppressing gcDNA synthesis. The anchored oligo-T primer has degenerated nucleotide(s) at 3′ end to determine cDNA priming at single position per transcript^9^,^10^. Non-anchored oligo-T primers anneal randomly along 150–250 poly-A nucleotide tail of mRNA^11^, producing multiple cDNA molecules from a single template mediated by strand replacement activity of reverse transcriptase (RT).

**Figure 1:**
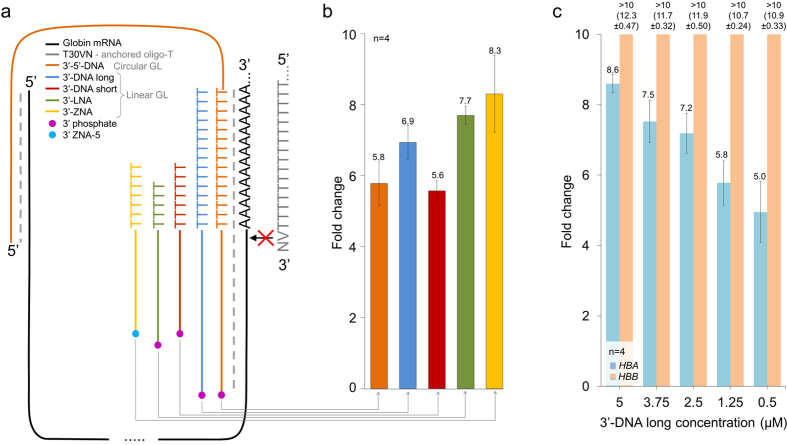
The nature and reduction effect of GlobinLock. (**a**) Schematic globin mRNA with possible masking oligonucleotides and anchored oligo-T primer. All oligo sequences and modifications are shown in Supplementary Table 1. (**b**) Five GL conditions and GL-negative controls were compared to measure globin α reduction fold change by qPCR. Template dilutions (10×) were used in this relative qPCR design and therefore the reduction effect up to ten is measured accurately according to existing dilution factor but fold change values above ten are out of the reported quantification range. (**c**) 3′-DNA long GL concentration effect on human globin α and β as measured by qPCR.

To discover the most effective and specific GL, multiple oligonucleotide variants that are complementary to human globin α and β were tested. Two types of GL oligonucleotides were designed: a linear GL masking the 3′ end of gmRNA and a circular GL masking the gmRNA 3′ with an additional binding site at the 5′ end to add specificity and to interfere with the opening of the gmRNA secondary structure. The natural shape of gmRNAs, where the 3′ and 5′ ends are in close proximity (Supplementary Figs 2 and 3) enabled the hypothetical formation of a circular gmRNA with a circular GL complex prior to cDNA synthesis (Fig. 1a).

The GLs were first evaluated by quantitative PCR (qPCR), measuring the yield change of α cDNA. *HBA* has predicted secondary structure at gmRNA 3′ end by two independent algorithms (Supplementary Figs 2 and 3). Therefore the predicted α self-binding compete with GL, indicating that sensitive test system should be used to ascertain efficient and stringent hybridization and oligonucleotide conditions for comprehensive gmRNA locking. To investigate stable binding and blocking properties of GL oligonucleotides, we tested locked nucleic acid^12^ (LNA) and zip nucleic acid^13^ (ZNA) modifications in addition to non-modified DNA oligonucleotides. In a parallel comparison, ZNA-modified GL provided the highest gcDNA reduction fold change (8.3 ± 1.1) and was more efficient than 3′-LNA and 3′-DNA long oligonucleotides (Fig. 1b). As the predicted secondary structure of α masks the gmRNA 3′ end for GL hybridization, the blocking is highly dependent on GL concentration, providing the highest fold change at the 5 μM level (Fig. 1c). Higher GL concentrations were not tested due to the possible risk of inhibition to immediate downstream cDNA synthesis in the same reaction tube.

**Figure 2:**
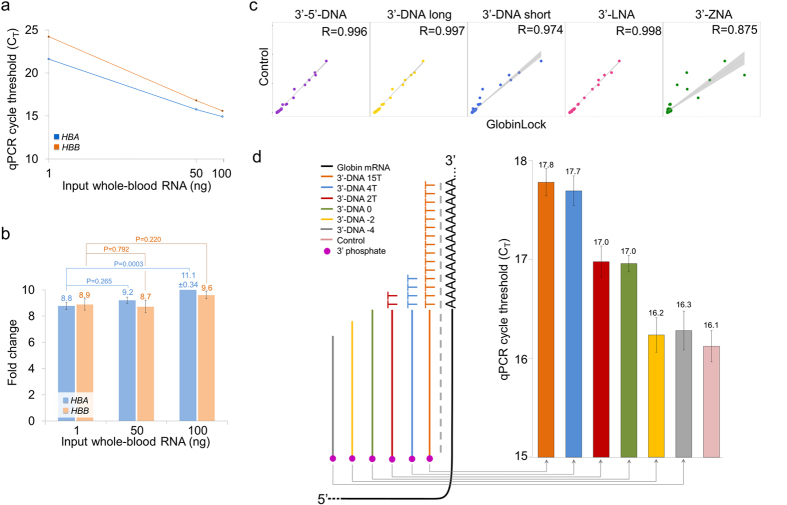
The effective range and limitations. (**a**) qPCR cycle threshold values are presented to indicate the number of required PCR cycles if less than 100 ng of whole-blood RNA is GL treated. (**b**) GL fold change reduction effect was measured by qPCR for three whole-blood RNA inputs: 1, 50 and 100 ng. (**c**) Scatter plot to measure the GL specificity based on an ERCC 92 mRNA spike-in mix over five different GL oligonucleotides compared with a GL-negative control. (**d**) The importance of the GL T_n_ tail for masking. Different GL oligonucleotides with variable lengths of T_n_ tails were analyzed, and the reduction effect of globin α was compared with the GL-negative experiment using qPCR.

Despite the micromolar concentration of GL, the specificity in gcDNA reduction was verified by the addition of artificial spike-in molecules at 1, 50 and 100 ng of input wbRNA. The spike-in is pre-formulated set of 92 polyadenylated artificial mRNA-like molecules at different concentrations to enable up to date expression data normalization. In GL specificity test, GL oligonucleotides had no binding sites on spike-in molecules, enabling to evaluate the GL’s specificity. As a result, we found no differences in spike-in detection (*P* ≥ 0.107, *n* = 3, Student’s *t*-test, Supplementary Table 2) at different wbRNA concentrations in the presence of GL, indicating that only up to 10 additional PCR cycles were required to analyze 1 ng of wbRNA instead of 100 to compensate for the lower input material (Fig. 2a). Without RNA-seq proofs, the theoretical wbRNA input requirement for GL is >10 times less than for GLOBINclear^TM^ or Globin-Zero^TM^ kits, as reviewed in Supplementary Fig. 1. In addition to specificity, GL efficiency was evaluated at 1–100 ng wbRNA range by qPCR. As a result, α and β reduction fold change increased slightly using higher amount of input RNA in this test system (Fig. 2b).

The specificity of GL was measured also by RNA-seq where different GL conditions were compared with GL negative samples. For all GL oligos, the spike-in experiment indicated high correlations. Still in 3′-LNA (R = 0.998) and 3′-DNA long GL (R = 0.997) higher specificity was seen than using 3′-DNA short GL (R = 0.974) and 3′-ZNA (R = 0.875) (Fig. 2c).

The presence of the GL oligo T-tail is critical for efficient masking of gmRNA. The minimum length of T-tail was verified by experiments where gradually shorter oligonucleotides were compared. The GL with T_15_ was used as a control and compared with the T_4_, T_2_, and T_0_ GL oligonucleotides. A minimum of 4 T nucleotides was required to enable a stable masking complex prior to T_30_VN hybridization (Fig. 2d). It has been shown that once initiated, gcDNA synthesis can be inhibited only by peptide nucleic acid oligonucleotides^14^ (PNA), as any other downstream hybridized DNA or LNA molecules are removed by the strand-displacement activity of RT^15^.

The global GL effect for seven wbRNAs and one negative control over all conditions was analyzed using a modified single-cell tagged reverse transcription RNA-seq method^9^,^16^ (STRT). The STRT uses oligo-T priming similar to advanced SMART-Seq protocols^17^, G&T-seq^18^ and CEL-seq^19^ protocols. The wbRNA was diluted to 30 ng/μl, and 2 μl was treated for 10 min at 60 °C in the presence of GL in GL-K^+^ buffer prior to cDNA synthesis. The presence of K^+^ cation is essential for stringent GL hybridization and is harmless for downstream RT reaction in the same reaction tube. Alkaline pH ensures the stability of mRNAs even after the short denaturation and blocking. It is notable that brief denaturation at 95 °C is essential for GL mediated locking reaction due to the presence of K^+^, elevating probably the annealing temperature of target gmRNA molecules. Only constant GL hybridization time and temperature (10 min at 60 °C) was tested by RNA-seq experiment in this report. Based on the nature of gmRNA and our empirical data about high GL concentration dependence, GL hybridization seems to take place just after gmRNA denaturation and before gmRNA forms its natural closed structure. Therefore shorter locking incubation times are rationale to test by RNA-seq.

All different GL oligonucleotide reactions were compiled into one 48-plex RNA-seq library. The library was sequenced on one Illumina HiSeq2000 lane, obtaining 0.9–4.1 M qualified reads per sample according to the type of GL oligo (Supplementary Table 3). The read mapping rate to reference was 88–92%. Our applied STRT RNA-seq method^9^ determines the 5′ start site of poly-A transcript, indicating the possible RNA degradation. Therefore the mRNA 5′-capture rate (RNA integrity) in GL experiment was 80–85% for all conditions, showing that the proportion of the mapped and intact transcripts was not decreased after GL treatment (Supplementary Table 3).

**Figure 3:**
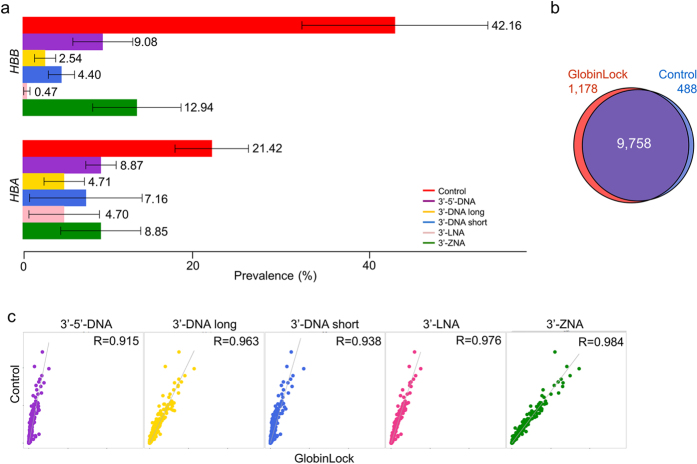
Reduction outcome. (**a**) Human globin α and β mRNA reduction fold change based on the normalized prevalence over all genes detected in RNA-seq, with or without GL (red). (**b**) Venn diagram showing the total number of genes detected with or without GL treatment, where GL treatment revealed 1,178 unique genes when compared with the control. (**c**) Whole-blood RNA transcriptome normalized read count comparing five different GLs with a GL-negative control.

RNA-seq without GL treatment revealed a 63.6% total prevalence of globin transcripts, with α and β contributing by 21.4% and 42.2%, respectively (Fig. 3a). Similar to our observations, surprisingly high variation in globin levels between studied samples was also described in previous report^4^, where globin prevalence differed between 52–76% over six samples. This difference is most likely representing the variable erythrocyte count in the original blood samples; the information which was not available for our study participants. β cDNA synthesis was reduced by ~90× (from 42.2% to 0.5%) by LNA-modified GL oligos, and α was reduced by ~4.5× (21.4% to 4.7%) by 3′-LNA or 3′-DNA long GL. However, the prevalence of α and β was reduced by >10×(63.6% to 5.2%) in a combination where 3′-LNA and 3′-DNA long or only 3′-LNA GLs were used for β and α, respectively. The modest reduction effect of α (4.5×) is explained by secondary structure of gmRNA. Compared with the β mRNA, in which the last 26–28 bp at the 3′ end are predicted to be free of self-assembling, potentially enabling efficient GL hybridization, α mRNA has a tight self-binding structure, making GL binding more competitive (Supplementary Figs 2 and 3).

Analyzing human wbRNA samples, 9,758 genes were detected in total. Without the GL treatment, 488 genes were uniquely detected (Fig. 3b). These genes showed normalized mean read count values at a frequency of 1 × 10^−5^, indicating the random and scarce appearance of the genes (Supplementary Table 4). The top 10 transcripts were analyzed by BLASTN at their 3′-ends, but no significant similarities with GL sequences were detected. A slightly higher gene detection rate in GL treatment compared with the GL-negative sample was expected^4^,^5^. The normalized read count (~1 × 10^−5^) revealed that the detection of an additional 1,178 genes (Fig. 3b) was random based on the low read count per transcript. The genes detected, as well as their normalized prevalence and 95% confidence intervals, are shown in Supplementary Table 5.

A normalized read count comparison of the five different GL oligonucleotides with the GL-negative control RNA samples revealed a R ≥ 0.915 correlation (Fig. 3c), indicating the correlation of the rest of the thousands of transcripts after removing α and β from the analysis.

The high prevalence of globin mRNA in blood samples is a technical limitation for expression analysis in all animal species. Therefore, we designed 3′-DNA long type GLs for cow, dog, rat, mouse and zebrafish. Zebrafish yield only 10–20 μl of blood, making the amount of blood a limiting factor for wbRNA studies. The efficiency of GL in all species was tested by qPCR, quantifying the yield of gcDNA. A > 5× fold reduction was detected in all species except for cow α, dog α and zebrafish β (Supplementary Fig. 4). Sanger re-sequencing of dog and zebrafish at the ultimate 3′ end of gmRNA suggested the existence of a possible additional motif (AGCCT, Supplementary Table 6) in dog α1. Simultaneously, no dog α2 was detected. This finding may be explained by the 84% GC content in the last 50 bp of the gmRNA region that could potentially suppress simultaneous α1 and α2 amplification prior to cloning. Three of 10 independent clones identified a possible TAAAAGC motif deletion in zebrafish β. We suggest that high-quality re-sequencing of globin mRNAs, in particular at the most 3′ downstream region, may be needed for GL assay design in many model organisms.

In conclusion, here we present a simple and cost-effective method to overcome globin mRNA caused limitation in biomarker research when whole-blood RNA samples are used. Presented data obtained by qPCR and RNA-seq proofs the GL positive reduction effect with adult human whole-blood RNA samples and indicates its potential for species as mouse, rat, cow, dog and zebrafish.

## Methods

### Ethics statements

The study protocol of human samples was approved by the Research Ethics Committee of the University of Tartu (221/M-31). Written informed consent was obtained from all analyzed persons. All experiments were performed in accordance with relevant guidelines and regulations.

All methods with animal blood samples were performed in accordance with the European Communities Directive (86/609/EEC) guidelines and regulations. The used experimental protocols were approved by Animal Clinic of the Estonian University of Life Sciences (Estonian Veterinary and Food Board (KL1203)), the Animal Ethics Committee of State Provincial Office of Southern Finland (ESAVI/6054/04.10.03/2012) and the Stockholm North Experimental Animal Committee (N128/15).

### RNA secondary structure prediction

Human RefSeq *HBA1* (α), *HBA2* (α), and *HBB* (β) mRNA sequences with additional A_5_ nucleotides at 3′ to mimic the poly-A strand were used to predict secondary structures. Two structure prediction software packages, *mfold v3.6*^20^ and *The Vienna RNA Websuite*^21^, were used (Supplementary Figs 2 and 3).

### GlobinLock design

The GL molecule is a single strand DNA (Sigma Aldrich), LNA (Exiqon) or ZNA (Metabion) oligonucleotide with 3′ modification to block possible 3′ extension during enzymatic reactions. All oligonucleotides are shown in Supplementary Table 1. Based on the gmRNA sequence, two types of GL molecules were designed. The circular GL (3′-5′-DNA, Fig. 1a) has two complementary sequences to gmRNA. The 5′ end of circular GL binds to the mRNA 5′ end, covering 22 bp of α and 30 bp of β. The circular GL 3′ end binds to the gmRNA 3′ end to 28 bp of α and 31 bp of β to achieve similar T_m_ over all the specific regions. The circular GL has a 15 bp poly-T tail to bind and flank the 5′ end of the mRNA poly-A. The poly-T, together with the GL specific upstream region, form the core of the GL oligo that flanks the binding site of the anchored oligo-T primer (T_30_VN) prior to RT. A nonspecific linker with a variable length (55 bp of α and 44 bp of β) is used to connect the GL oligo 5′ and 3′ specific regions and to form a circular complex between GL and gmRNA. The linear GL (3′-ZNA/LNA/DNA short and long GL) were designed to mask only the T_30_VN priming site. The linear GL oligos contain up to 30 bp at the 3′ end that are specific to the gmRNA 3′ protein coding end and a ≤15 bp poly-T stretch complementary to the mRNA poly-A tail. The 3′ end of linear GL is blocked by phosphate to prevent enzymatic extension. The linear GL binds only at the 3′ site of gmRNA, forming no circular complex of GL and gmRNA.

### Blood sampling and RNA extraction

Blood samples from humans (*n* = 7), cows (*n* = 3), dogs (*n* = 2), rats (*n* = 3), and mice (*n* = 6) were collected into PAXgene Blood RNA tubes (PreAnalytiX). The wbRNA of zebrafish (10–20 μl) was collected and extracted immediately by the TRIzol method. For species other than mouse and zebrafish, 2.5 ml of blood was collected. For mouse samples, blood samples from several animals were pooled to obtain the required (2.5 ml) volume. All PAXgene samples were incubated for 24 h at room temperature prior to PAXgene Blood RNA Kit extraction, according to the manufacturer’s instructions. RNA concentrations and the RNA integrity number (RIN) were measured with an Agilent 2100 Bioanalyzer Total RNA Nano kit (Agilent Technologies) or Qubit Fluorometer (Thermo Fisher Scientific).

### GlobinLock efficiency by quantitative PCR (qPCR)

RT was performed on all RNA samples for subsequent quantification with qPCR. During the cDNA synthesis, various GL molecules for different species or modifications (Supplementary Table 1) were added to the reaction mix to inhibit RT of α and β gmRNA molecules. Another reaction mix contained nuclease-free water instead of GL and served as a negative control and a comparison for determining the oligonucleotide masking effect in qPCR. Globin mRNA masking reactions were then carried out using GL-K^+^ buffer containing 1 μl of 50% PEG-6000 (Sigma Aldrich), 1.5 μl of 3.2 M betaine (Sigma), 0.4 μl of 25 mM dNTP mixture (Thermo), 0.375 μl of 2 M KCl (Sigma), 0.05 μl of 1 M Tris-HCl (Sigma, pH 8.0) and 0.05 μl of 10% Triton X-100 (Sigma) per blocking reaction. For α and β globin blocking, 0.12 μl of each specific 3′-DNA long GL oligonucleotide (100 μM) was added to each reaction, and an equal amount of nuclease-free water was used for negative controls. Nuclease-free water was added to obtain a final volume of 4 μl.

Two microliters of wbRNA (20 ng/μl) were added to 4 μl of GL-K^+^ buffer and kept on ice until denaturation. The gmRNA masking and cDNA synthesis reactions were performed in 0.2-ml tubes using common thermocyclers. The reaction conditions were as follows: 95 °C for 30 s for initial denaturation, 60 °C for 10 min for GL oligo hybridization and a 42 °C hold for the loading of 5 μl of RT mixture. The 5 μl of RT mixture contained 2 μl of nuclease-free water, 0.04 μl of 100 μM T_30_VN, 1.6 μl of 3.2 M betaine, 0.5 μl of 1 M Tris-HCl (pH 8.0), 0.075 μl of 1 M MgCl_2_ (Sigma), 0.5 μl of 100 mM DTT (Sigma), 0.18 μl of RiboLock RNase Inhibitor (Thermo), and 0.13 μl of RevertAid Premium Transcriptase (Thermo). The concentrations were calculated for the final RT volume of 10 μl, including the GL-K^+^ buffer. Cycling parameters continued at 42 °C for 60 min for the RT reaction and 85 °C for 5 min for RT inactivation. Quantitative PCR was conducted using HOT FIREPol EvaGreen qPCR Mix Plus (ROX) (Solis BioDyne) with a 7500 Fast Real-Time PCR instrument (Applied Biosystems) according to the manufacturer’s instructions; 200 nM primers and 1 μl of template were used in a 20 μl reaction volume. The primers were designed using Primer3 v4.0.0 software^22^. The generation of specific products was ensured by performing a melting curve analysis of the samples during the real-time PCR program. We also performed conventional PCR for all globin primers and analyzed the products with 2% TAE buffered agarose gel electrophoresis. All samples were also run with 10× diluted cDNA to enable quantification. The thermal cycling conditions were as follows: 95 °C for 15 min for activation and then 30 cycles of 95 °C for 15 s, 62 °C for 20 s, 72 °C for 30 s (at the end of which the fluorescence was measured). The qPCR data were analyzed using Applied Biosystems 7500 Software v2.0.5.

### GlobinLock effect by RNA-seq

The modified STRT method was used. Human wbRNA samples were diluted with RNase-DNase-free water to a concentration of 30 ng/μl, and 2 μl was added to 4 μl of GL-K^ + ^buffer. The buffer contained 1 M betaine, 2 mM dNTP mixture, 10 mM Tris (pH 8.0), 150 mM KCl, 0.2% Triton X-100, 2 μM equimolar barcoded 48-plex template switching oligonucleotides, and 5 μM GL α and β oligonucleotides (Supplementary Table 1). The human wbRNA samples (*n* = 7) were placed in the 48-plex reaction plate, and each well was tagged for sequencing with an individual barcode. After mixing GL-K^+^ and RNA on ice, the RNA was denatured for 30 s at 95 °C and incubated for 10 min at 60 °C for GL masking and continued for 60 min at 42 °C. Just after the 60 °C incubation, the block was cooled to 42 °C, and 5 μl of RT mixture was added to initiate cDNA synthesis. The RT mixture contained 1 M betaine, 50 mM Tris (pH 8.0), 5 mM DTT, 7.5 mM MgCl_2_, RiboLock (0.7 U/μl), 400 nM T_30_VN and RevertAid Premium reverse transcriptase (7 U/μl). The concentrations were calculated for final RT in a volume of 10 μl, including the GL-K^+^ buffer. Two microliters of ERCC Mix 1 (Ambion), a 1:500 spike-in dilution with nuclease-free water, were used per whole 48-plex library. After a 60-min RT reaction at 42 °C and a 5 min inactivation of RT at 85 °C, the contents of all 48 reaction wells (~500 μl) were pooled into a 2.0-ml tube. Dynabeads MyOne C1 Streptavidin (Invitrogen, 100 μl) beads were washed twice and used to capture the formed cDNA molecules (and free primers) according to the instructions. After three rounds of EB buffer (10 mM Tris, pH 8.0) and one round of water washing, the DNA-enriched beads were suspended in 75 μl of water and incubated at 75 °C for 3 min to release biotin from the streptavidin beads. The supernatant was used as a template for further full cDNA amplification as described previously^16^. The purified cDNA pool was first amplified using 14 cycles of PCR followed by 15 additional cycles to introduce the complete sets of adapters for Illumina sequencing. The libraries were size-selected (200–400 bp) using the sequential AMPure XP bead selection protocol described previously^16^.

### RNA-seq data analysis

Preprocessing of the RNA-seq raw sequences, alignment and quantitation were performed using the STRTprep pipeline^16^ (https://github.com/shka/STRTprep). The pipeline reports only uniquely mapped reads. The two loci *HBA1* and *HBA2* are highly similar and led to unmappable reads. Therefore, the *HBA2* locus and the upstream 500 bp (chr16:222346-223709 on hg19 reference genome) were masked before alignment, resulting in the mapping of both *HBA1* and *HBA2* reads at *HBA1*. Branch v3dev (commit 698fa8c…) was used as the standard procedure with PCR-bias reduction based on the unique molecular identifier (UMI) and branch v3devNoUMI (commit e0d6721…) was a special procedure that skips the reduction step. The figures were generated using R version 3.2.2.

### GlobinLock design for other species and Sanger re-sequencing

In addition to human, the GL oligos were designed to mask gmRNA of cow, dog, rat, mouse, and zebrafish. For those species, qPCR-based quantification was used, and linear GL oligonucleotides were applied. The GL design followed the principle that the specific part has a T_m_ ~70 °C plus an additional 15 bp poly-T tail to stabilize the complex during T_30_VN priming and cDNA synthesis. The qPCR-based GL efficiency was detected as described above. Due to the inability to detect the GL effect with α of cow and dog, and β of zebrafish (Supplementary Fig. 4), the gmRNA 3′ ends were amplified from cDNA, cloned to a vector and Sanger re-sequenced to confirm the sequence of the studied transcripts. Briefly, the transcripts were amplified using primers indicated in Supplementary Table 1 and HotStarTaq DNA polymerase (Qiagen) according to manufacturer’s instructions. The products were amplified using a PCR program as follows: 95 °C for 5 min and 30 cycles of 95 °C for 30 s, 60 °C for 30 s, 72 °C for 30 s and final extension 72 °C for 5 min. PCR products were cloned into a pCRII-dual promoter TOPO vector using the TOPO TA cloning kit (Invitrogen) and sequences were verified by Sanger sequencing (Eurofins Genomics). The full Sanger sequences are available in Supplementary Table 7.

## Additional Information

**How to cite this article**: Krjutškov, K. *et al*. Globin mRNA reduction for whole-blood transcriptome sequencing. *Sci. Rep.* **6**, 31584; doi: 10.1038/srep31584 (2016).

## Supplementary Material

Supplementary Information

Supplementary Table 1

Supplementary Table 3

Supplementary Table 4

Supplementary Table 5

Supplementary Table 6

Supplementary Table 7
