# Supplementary material for: Globin mRNA reduction for whole-blood transcriptome sequencing
Source: Sci Rep. 2016 Aug 12;6:31584. doi: 10.1038/srep31584 (PMC4981843; doi:10.1038/srep31584)
Supplement: Supplementary Information [file srep31584-s1.pdf]

Globin mRNA reduction for whole-blood transcriptome sequencing

Kaarel Krjutškov[1-3], Mariann Koel[2;4], Anne Mari Roost[2], Shintaro Katayama[1], Elisabet Einarsdottir[1;3], Eeva-Mari Jouhilahti[1], Cilla Söderhäll[1;5], Ülle Jaakma[2;6], Mario Plaas[7], Liselotte Vesterlund[1], Hannes Lohi[3], Andres Salumets[2;7;8], Juha Kere[1;3]

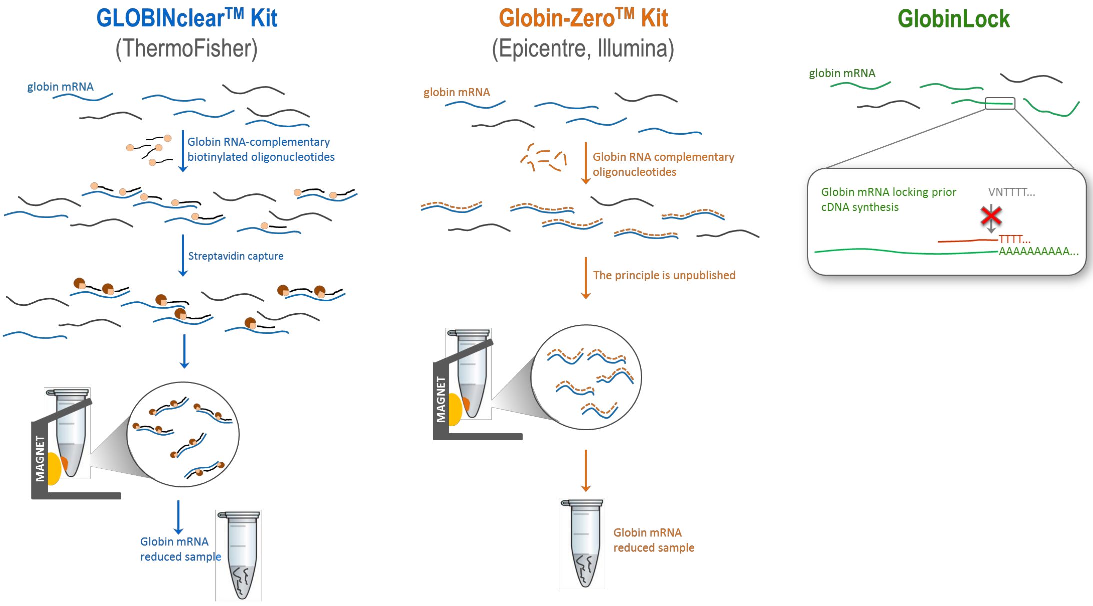

	GlobinClear™ Kit[1]	Globin-Zero™ Kit[2]	GlobinLock
Principle	Specific oligonucletoide hybridization and biotin-streptavidin magnetic capture	Specific oligonucleotide hybridization. The capture principle is not published	Specific oligonucleotide hybridization and globin mRNA blocking prior oligo-T priming
Isolation technology	Magnetic capture	Magnetic capture	Specific blocking
Need of previous DNase I treatment	No	Yes	No
Enzymatic treatment during capture	No	No	No
Globin reduction rate	>95%	>98%	>91%[3]
Need of additional equipment	Magnetic stand, hybridization block	Magnetic stand, hybridization block	No
Starting material (total blood RNA, µg)	1 - 10	1 - 5	0.01 - 0.1
Input concentration (ng/µl)	≥70	≥38	≥10
Purification time (min)	90	90	10
Downstream cDNA synthesis by random primer	+	+	-
Downstream cDNA synthesis by oligo-T primer	+	+	+
Purification before downstream application	Yes	Yes	No
Price per single reaction[4] (USD)	35	96	0.5[3] - 4.5[5]
High-throughput compatible	Yes	No	Yes
PubMed references (PubMed ID)	17065423, 22257641, 20477359, 17303101	NA	NA

[1] www.thermofisher.com/order/catalog/product/AM1980
[2] support.illumina.com/sequencing/sequencing_kits/globin-zero-gold.html
[3] GlobinLock LNA oligonucleotides
[4] manufacturer's suggested retail price in Sweden
[5] circular GlobinLock oligonucleotides

Supplementary Figure 1. Comparison of two commercially available globin mRNA reduction assays (GLOBINclear™ and Globin-Zero™) based on manufacturer's product information from official webpage. In addition, GlobinLock principle and parameters are depicted.

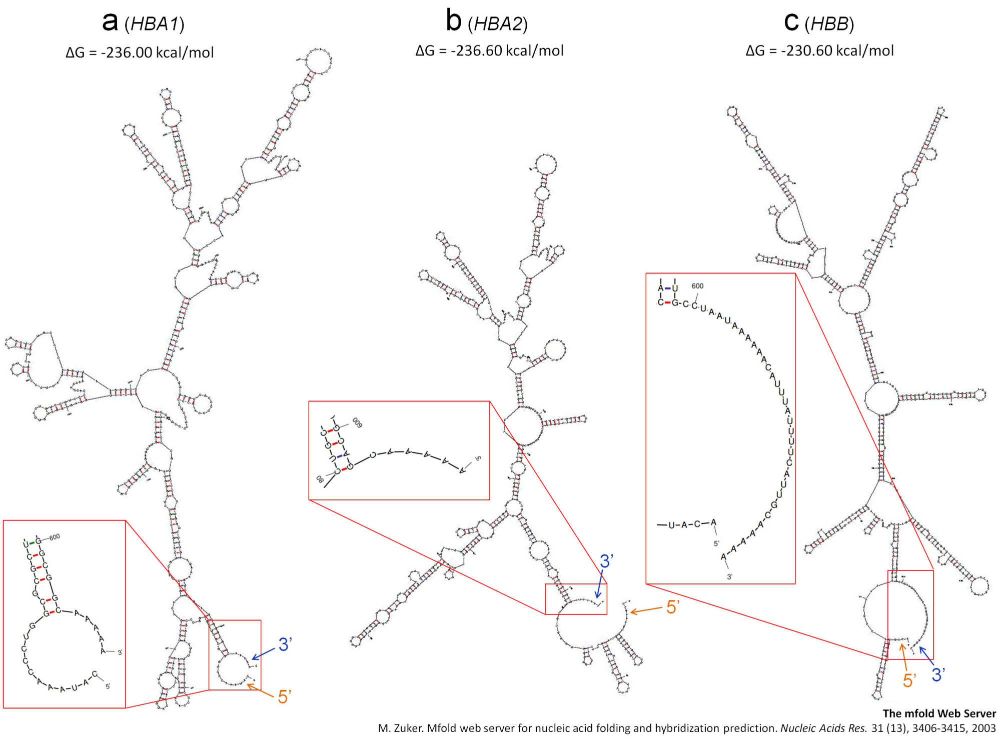

Supplementary Figure 2. Human globin mRNA secondary structure prediction. The Mfold web server was used to (**a**) predict human globin α1 (*HBA1*), (**b**) human globin α2 (*HBA2*) and (**c**) human globin β (*HBB*) self-folding. The delta G value and the globin zoom-in at the 3'-5' proximity region are depicted to visualize the binding site of GlobinLock or anchored oligo-T primer.

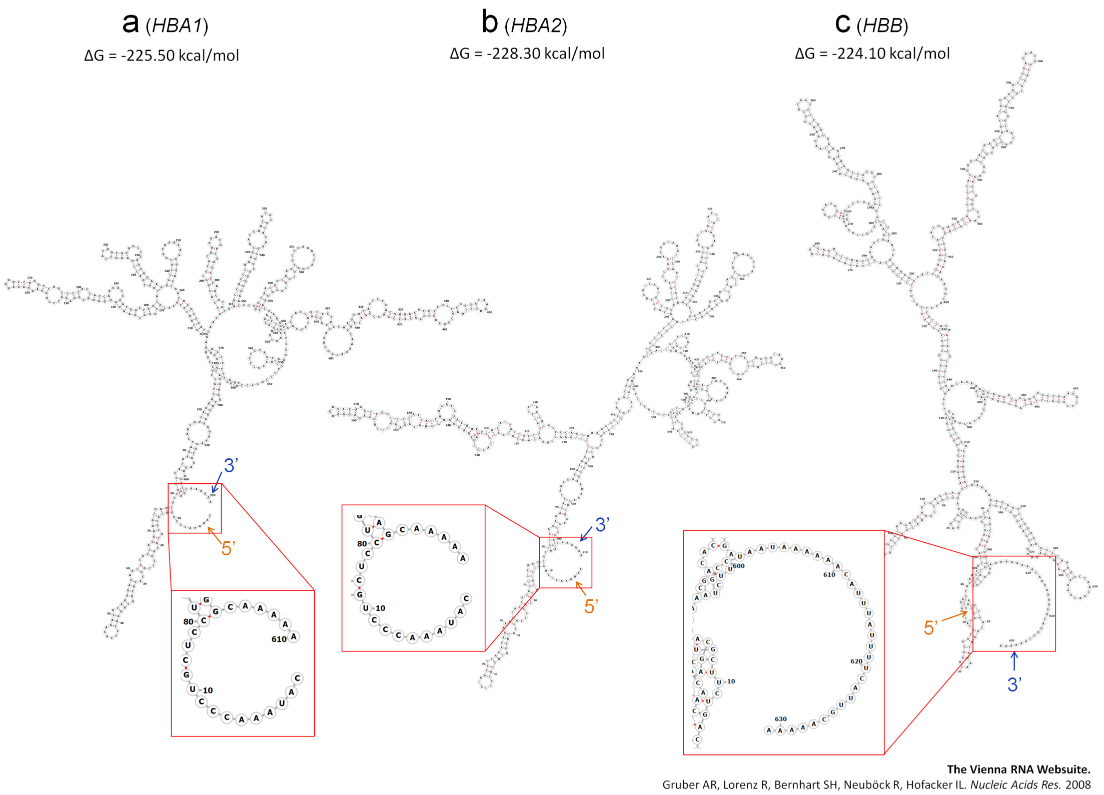

Supplementary Figure 3. Human globin mRNA secondary structure prediction. The Vienna RNA websuite was used to (**a**) predict human globin α1 (*HBA1*), (**b**) human globin α2 (*HBA2*) and (**c**) human globin β (*HBB*) self-folding. The delta G value and the globin zoom-in at the 3'-5' proximity region are depicted to visualize the binding site of GlobinLock or anchored oligo-T primer.

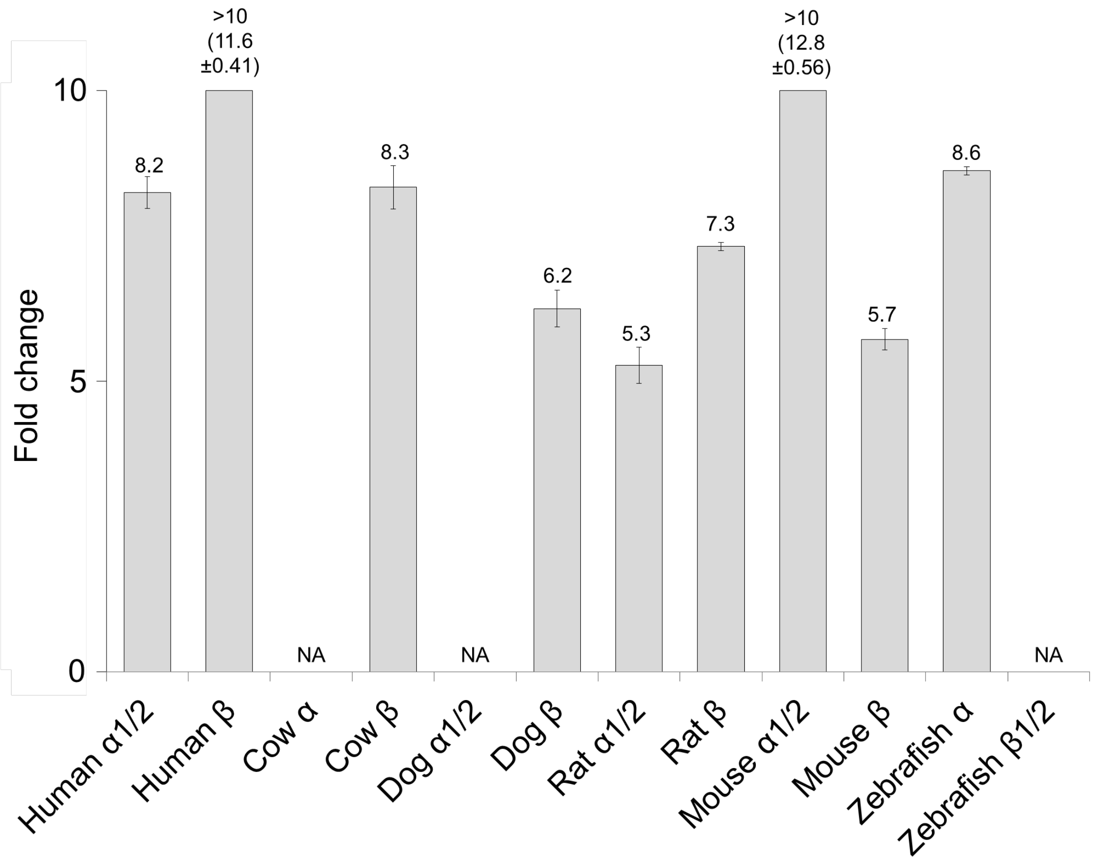

Supplementary Figure 4. The prevalence of globin mRNAs from different species was reduced by type 3'DNA long GlobinLock oligonucleotides, quantified by qPCR. NA indicates the inability to detect the specific globin with unique primers using a SYBR green qPCR assay. Template dilutions (10×) were used in this relative qPCR design and therefore the reduction effect up to ten is measured accurately according to existing dilution factor but fold change values above ten are out of the reported quantification range.

Supplementary Table 1. Used GlobinLock oligonucleotides with specific modifications and purifications. In addition, qPCR primers, barcoded 48-plex template-switching oligonucleotides for 48-plex RNA-seq experiment and globin cloning primers of different species are listed.

>>> *Attached as a separate file "Suppl Table 1"*

Supplementary Table 2. The specificity of GlobinLock with 1–100 ng of whole-blood RNA, using two artificial spike-in molecules. One-way ANOVA analysis together with *t*-test *p*-values to measure the significance based on two spike-in molecule (Spike-1 and Spike-2) detection rates at different whole-blood RNA input levels.

**Spike-1**					*t*-test p-value		
GlobinLock					+	+	+
	Blood-RNA input				1 ng	50 ng	100 ng
		C_T values			24.827	24.873	24.772
					24.743	24.844	24.761
					24.72	24.766	24.709
+	1 ng	24.827	24.743	24.72	*NA*	*0.2310*	*0.6945*
+	50 ng	24.873	24.844	24.766	*0.2310*	*NA*	*0.0982*
+	100 ng	24.772	24.761	24.709	*0.6945*	*0.0982*	*NA*

P=0.1910 by one-way ANOVA

**Spike-2**					t-test p-value		
GlobinLock					+	+	+
	Blood-RNA input				1 ng	50 ng	100 ng
		C_T values			24.265	24.119	24.588
					24.311	24.244	24.195
					24.324	24.250	24.206
+	1 ng	24.265	24.311	24.324	*NA*	*0.1077*	*0.8312*
+	50 ng	24.119	24.244	24.250	*0.1077*	*NA*	*0.4092*
+	100 ng	24.588	24.195	24.206	*0.8312*	*0.4092*	*NA*

P=0,5404 by one-way ANOVA

Supplementary Table 3. The statistics for GL RNA sequencing results over five GL conditions and the control. RNA-seq data QC parametres from mapping (No. of raw reads per sample (average)) to (mRNA 5'-end capture rate (%)). The data were analyzed with unique molecular identifier (UMI) correction and without. The results described in the manuscript are without UMI correction. The analytical pipeline details are described in Methods and https://github.com/shka/STRTprep

>>> *Attached as a separate file "Suppl Table 3"*

Supplementary Table 4. Uniquely detected genes without different GlobinLock oligonucletoides. The top 10 uniquely expressed genes under different GlobinLock conditions and the GlobinLock-negative control. The mRNA's 3'-most sequences are depicted and

compared with GlobinLock sequences to find possible interactions. BLASTN scores indicated very low specificity because the values remained <33 in a 100 scale.
>>> *Attached as a separate file "Suppl Table 4"*

Supplementary Table 5. The detected genes with normalized prevalence and 95% confidence intervals. The "Type" represents the type of tested GlobinLock oligonucleotides or negative control.
>>> *Attached as a separate file "Suppl Table 5"*

Supplementary Table 6. Alignment of high-quality Sanger re-sequencing of dog and zebrafish clones compared to the reference and GlobinLock molecule. Novel motifs or deletions are marked in red.
>>> *Attached as a separate file "Suppl Table 6"*

Supplementary Table 7. The high-quality raw data of Sanger re-sequencing.
>>> *Attached as a separate file "Suppl Table 7"*
